# Notes of a Case of Impaction of the Appendix Vermiformis—Cœcitis—Death—Post-Mortem Appearances

**Published:** 1862-01

**Authors:** De Laskie Miller


### NOTES OF A CASE OF IMPACTION OF THE APPENDIX VERMIFORMIS—CŒCITIS—DEATH —POST-MORTEM APPEARANCES.

By DE LASKIE MILLER, M. D.

The following brief history of a case which recently came under my care, is so strikingly similar in its course and termination to cases which occasionally occur in practice, in which treatment proves unavailing, but in which no post-mortem examination is made, which would reveal the real cause of the disease and death, that I am induced to give it publicity. It may furnish a key for the explanation of similar cases, which, like this, cannot be regarded otherwise than unfortunate in the extreme, on account of the distressing symptoms, and the utter impossibility of arresting the progress of the disease or of averting the sad termination.

Walter B., aged about twenty months, rather over the usual size of children of his age, had enjoyed good general health, with the exception of occasional attacks of slight indisposition. A somewhat protracted course of psorophthalmia from which he had recovered gave rise to a suspicion of strumous predisposition.

In the early part of July I was requested to visit him on account of a slight derangement of the bowels. He had some diarrhœa, though the discharges were not very frequent or profuse, and although he suffered from occaasional attacks of pain, there was no appearance of any unusual complication which should render the case unmanageable. Antacid astringents and anodynes were prescribed which afforded temporary relief. The paroxysms of pain, however, continued to recur with increasing severity; the evacuations from the bowels ceased, but nausea and vomiting ensued. The abdomen now began to be distended and tympanitic. A congenial embilical hernia gave rise to the suspicion that strangulation might cause some of the symptoms, but a careful and thorough examination, in which I obtained the valuable assistance of Prof. Brainard, led to the conclusion that no complication of this kind existed. The bowels not having acted for several days, an attempt was made to effect this and overcome any obstruction which might exist, by large enemas of warm water introduced through one of O'Brien's tubes, This was repeated from time to time, without, however, producing the desired effect. The paroxysms of pain continued to increase in severity until they became agonizing, and the vomiting was more frequent and distressing. These symptoms continued, while the strength of the system gave way, notwithstanding my most sedulous endeavors to sustain the vital powers, and death took place twelve days after my first visit.

The post-mortem examination made twelve hours after death, in which I was assisted by Prof. Ingals, revealed the small intestines greatly distended, principally with gas, (but not entirely); the large intestines nearly empty. The coats of the bowels were but slightly injected and not discolored, except at the cæcum, the coats of which were considerably thickened and of a dark color. The appendix was enlarged, and completely filled and distended with fecal and decomposing matters. Its coats were thickened and of a livid color, and a few small patches of lymph lay on the peritoneal coat of it and the cæcum; with this exception the peritoneum appeared quite healthy. At the junction of the small with the large intestines two excrescences of considerable size were found, which were so situated as to have had the effect apparently of compressing and closing the ileo cæcal orifice.

The examination revealed the exact seat of the disease, the cause of its persistence and fatality, viz: the distension of the appendix with highly irritating matter. The cause of this impaction, or rather the reason why it does not more frequently occur so as to lead to a fatal termination, would be an interesting question for investigation.

The following selections, taken from the standard authors at hand, give the views entertained upon the causes, symptoms and termination:

"Diminished contractile power of the muscular coat, with distension of the intestine and over-exertion are the probable causes of propulsion of fæces into the appendix. * * However produced any concretion in this part leads to very serious results. * * In strumous patients these concretions more readily tend to an unfavorable result, leading to perforation, abscess or peritonitis. * * There appears to be a greater tendency to this local enteritis of the cæcum in strumous than in other subjects. * * Disease af the appendix sometimes exists for some time without manifesting any symptoms."—*Habershon on the Alimentary Canal.*

This author gives numerous cases where the fatal result was attributed to impaction of fæces in the appendix, and expresses the opinion that this is most liable to occur in early life.

"Whatever the seat of the obstruction its existence is portrayed by much the same train of symptoms in all cases."—*West on the Diseases of Children.*

Then follows an examination of the symptoms almost exactly as they occurred in the subject of this case.

"Not a few cases are recorded where the most violent inflammation was induced by the impaction of fœcal matter in the appendix, followed by gangrene of the tube and general peritonitis."—*Gross, Path. Anat.*

"This disease has been almost invariably fatal."—*London Lancet.*

Bell and Stokes conclude their description of the disease as follows:

"It will be observed that in the cases now related there was at first diarrhœa, which was followed by obstinate constipation. Such is the usual occurrence whenever the cellular substance round the cæcum becomes inflamed, owing to the loss of contractility in the muscular coat of the intestines by inflammation and partly to the mechanical pressure of the swelling on the cæcum, and on the colon also, and small bowels. * * The prognosis is by no means favorable."

Copland's Dictionery of Medicine contains a most elaborate and valuable article upon this subject, wherein it is stated that inflammation of the appendix, when occasioned by impaction, often does not extend to the cæcum even, but that such cases are most rapid and dangerous." In regard to treatment, it is advised that "the assiduous administration of enemata must not be neglected; it is entirely by their agency in this state of disease that the bowels are to be evacuated."

Similar views are expressed in Wood's Practice, in Dunglison's Practice, in the Library of Practical Medicine, etc., etc.

From the history of the case given above, and these references to the disease, the conclusion is irresistable, that if post-mortem examinations were made more frequently, this complication would be met with many times, where now it is not suspected.

